# Centrifugal Innervation of the Olfactory Bulb: A Reappraisal

**DOI:** 10.1523/ENEURO.0390-18.2019

**Published:** 2019-02-07

**Authors:** Estelle E. in ’t Zandt, Hillary L. Cansler, Heather B. Denson, Daniel W. Wesson

**Affiliations:** 1 Department of Pharmacology and Therapeutics; 2 Center for Smell and Taste, University of Florida, Gainesville, FL 32610

**Keywords:** centrifugal innervations, olfactory bulb, olfactory system, olfactory tubercle

## Abstract

The inter-regional connectivity of sensory structures in the brain allows for the modulation of sensory processing in manners important for perception. In the olfactory system, odor representations in the olfactory bulb (OB) are modulated by feedback centrifugal innervation from several olfactory cortices, including the piriform cortex (PCX) and anterior olfactory nucleus (AON). Previous studies reported that an additional olfactory cortex, the olfactory tubercle (OT), also centrifugally innervates the OB and may even shape the activity of OB output neurons. In an attempt to identify the cell types of this centrifugal innervation, we performed retrograde tracing experiments in mice utilizing three unique strategies, including retrobeads, retrograde adeno-associated virus (AAV) driving a fluorescent reporter, and retrograde AAV driving Cre-expression in the Ai9-floxed transgenic reporter line. Our results replicated the standing literature and uncovered robustly labeled neurons in the ipsilateral PCX, AON, and numerous other structures known to innervate the OB. Surprisingly, consistent throughout all of our approaches, no labeled soma were observed in the OT. These findings indicate that the OT is unique among other olfactory cortices in that it does not innervate the OB, which refines our understanding of the centrifugal modulation of the OB.

## Significance Statement

The perception of our environment relies on the distribution of sensory information throughout brain regions. This is true in the olfactory system wherein projections between olfactory centers, including feedback centrifugal input to the olfactory bulb (OB), provide the basis for olfactory perception. Here, we show that one olfactory cortical structure, the olfactory tubercle (OT), is unique among olfactory cortices in that it lacks feedback projections to the OB. This “negative result” is important in that it refines current models for the circuitry of our olfactory system and challenges previous literature reporting such a pathway in fact exists.

## Introduction

In our sensory systems, the initial steps of information processing are directed not only by bottom-up sources from the environment, but also by top-down inputs from higher-order structures (Kiselycznyk et al., 2006; Gilbert and Li, 2013; Terreros and Delano, 2015). This type of centrifugal modulation allows early sensory representations to be shaped by factors such as learning, and internal states including hunger and arousal (Lavin et al., 1959; Gervais and Pager, 1979; Sullivan et al., 1989; Wachowiak et al., 2009; Fletcher and Chen, 2010; Kato et al., 2012; Ross and Fletcher, 2018). Feedback projections may arise from neuromodulatory loci as well as cortical structures, and thus can have a wide range of effects on sensory representations and perception, including state-dependent sensory gating and experience-dependent plasticity (Shepherd, 1972; Petzold et al., 2009; Bajo et al., 2010; Devore and Linster, 2012; Smith et al., 2012; Rothermel et al., 2014; Terreros and Delano, 2015; Ogg et al., 2018). Major questions remain regarding the neural circuitry underlying centrifugal modulation.

In the olfactory system, odors detected in the epithelium are first processed in the olfactory bulb (OB; Schoppa and Urban, 2003; Ache and Young, 2005; Wachowiak and Shipley, 2006). Next, OB principal neurons called mitral and tufted cells convey information to one of several olfactory cortices, including the anterior olfactory nucleus (AON), piriform cortex (PCX), and the olfactory tubercle (OT), each of which is thought to play a specialized role in odor processing (Scott et al., 1980; Haberly, 2001; Brunjes et al., 2005; Gottfried, 2010; Wesson and Wilson, 2011; Wilson and Sullivan, 2011). Additionally, all of these structures are reported to send centrifugal inputs back to the OB (Shafa and Meisami, 1977; Kiselycznyk et al., 2006; Markopoulos et al., 2012; Rothermel and Wachowiak, 2014; Otazu et al., 2015), suggesting that each may contribute, perhaps in unique manners, to the modulation of early odor representations. For example, AON inputs to the OB are activated in odor-specific and state-dependent manners (Rothermel and Wachowiak, 2014). These AON inputs directly activate mitral and tufted cells and indirectly drive local inhibitory circuits, resulting in a widespread inhibition which is proposed to aid in suppressing OB background activity (Markopoulos et al., 2012). Similarly, activation of PCX inputs to the OB during odor stimulation enhances odor-evoked inhibition of mitral and tufted cells, though this occurs via different elements of the OB microcircuit (Boyd et al., 2012; Markopoulos et al., 2012). Further, PCX inputs to the OB target mitral cells, but not tufted cells (Otazu et al., 2015). Together these results indicate that feedback projections from the PCX and AON may be poised to impact odor perception, perhaps differentially.

The OT is an olfactory cortical structure situated within the ventral striatum (Alheid and Heimer, 1988; Wesson and Wilson, 2011). Previous work has described its roles in odor processing (Wesson and Wilson, 2010; Payton et al., 2012; Carlson et al., 2014, 2018; Xia et al., 2015), odor hedonics (Agustín-Pavón et al., 2014; FitzGerald et al., 2014; Gadziola et al., 2015; Murata et al., 2015; Howard et al., 2016; Zhang et al., 2017a), and motivated behavior (Prado-Alcala et al., 1984; Ikemoto, 2003; Sellings et al., 2006; Agustín-Pavón et al., 2014; FitzGerald et al., 2014; DiBenedictis et al., 2015; Gadziola and Wesson, 2016). The OT receives input from multiple olfactory structures, including the OB, AON, and PCX, as well as numerous brain regions important for affect, motivation, and cognition (White, 1965; Schwob and Price, 1984; Zahm and Heimer, 1987; Cleland and Linster, 2003; Ikemoto, 2007; Wesson and Wilson, 2011; Zhang et al., 2017b). OT targets include a similarly wide array of structures, with its principle output neurons being medium spiny neurons that innervate basal ganglia as well as additional structures (Wesson and Wilson, 2011; Zhang et al., 2017b). Notably, some studies have concluded, based on tracing methods or electrophysiological recordings, that the OT innervates the OB (Heimer, 1968; Shafa and Meisami, 1977; Gervais, 1979; Zhang et al., 2017b).

We set off with the goal to explore the organization, cell types, and functional role of these reported OT projections to the OB. To do so we employed tracing methods in non-transgenic mice and in a floxed reporter line. Ultimately, all the tracing methods we used failed to reveal OT projections to the OB, suggesting that the OT does not centrifugally contribute to early olfactory processing, in contrast with previous reports.

## Materials and Methods

### Animals

**Figure 1.**
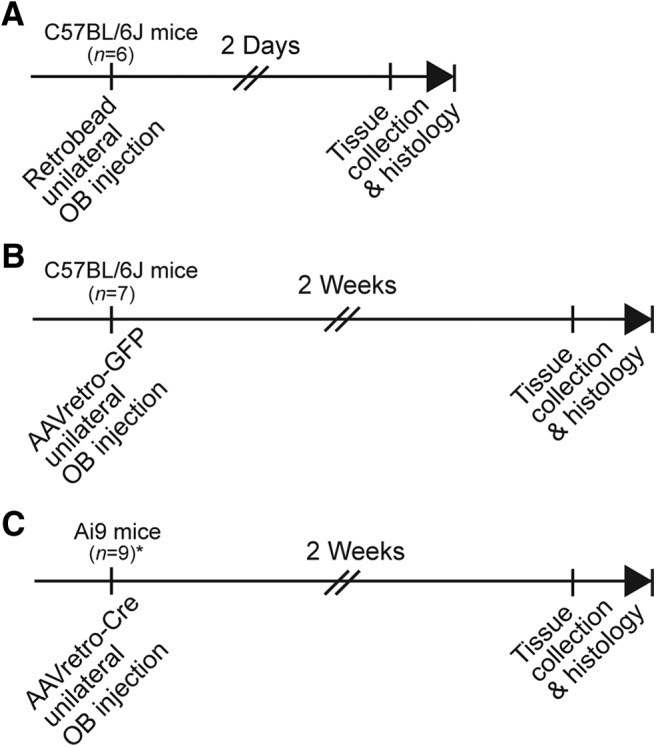
Timeline of methods and experimental groups. Three groups of mice were used in three different experimental paradigms. ***A***, C57BL/6J mice received retrobead injections into the OB and were perfused at 2 d. ***B***, C57BL/6J mice received AAVretro-GFP injections into the OB and were perfused at two weeks. ***C***, Transgenic Ai9 reporter mice received AAVretro-Cre injections and were perfused at two weeks; *three of nine Ai9 mice received multiple injections of AAVretro-Cre throughout one OB (see Materials and Methods; Table 1).

Three different experimental approaches were used (Fig. 1). Two used C57BL/6J mice (bred in University of Florida vivarium from breeder stock originating from The Jackson Laboratory), and one used Cre-dependent reporter mice Gt(ROSA)26Sor^tm9(CAG-tdTomato)Hze^ (Madisen et al., 2012) obtained from Jackson Labs (“Ai9”; stock #007905, The Jackson Laboratory). First, C57BL/6J mice received unilateral OB injections of red retrobeads (Lumafluor, Inc.) following surgical procedures described below and were perfused 2 d later (Fig. 1*A*). Second, C57BL/6J mice received unilateral OB injections of the adeno-associated virus (AAV) pAAV-hSyn-EGFP (“AAVretro-GFP”; Addgene viral prep #50465-AAVrg) and were perfused two weeks later (Fig. 1*B*). Additional mice received OT injections of AAVretro-GFP to confirm that the virus used was capable of infecting OT neurons and were perfused one week later. Finally, homozygous Ai9 mice received unilateral OB injections of pAAV-Ef1α-mCherry-IRES-Cre (“AAVretro-Cre”; Addgene viral prep #55632-AAVrg) and were perfused two weeks later (Fig. 1*C*). Again, additional Ai9 mice received OT injections of AAVretro-Cre to confirm that the virus used was capable of infecting OT neurons and were perfused one week later.

All mice were 6–12 weeks old (*n* = 19 male, *n* = 4 female). All animal procedures were in accordance with the guidelines of the National Institutes of Health and were approved by the Institutional Animal Care and Use Committee at the University of Florida. Mice were housed in groups on a 12/12 h light/dark cycle with *ad libitum* access to food and water.

### Surgical procedures

Mice were anesthetized with ∼3% isoflurane in 1 l/min O_2_ and mounted into a stereotaxic frame, equipped with a heating pad to maintain body temperature at 38°C. Depth of anesthesia was confirmed by lack of toe-pinch response and meloxicam analgesic was administered subcutaneously (5 mg/kg; Putney, Inc.). After removing fur, the scalp was cleaned using betadine followed by 70% ethanol. Subcutaneous marcaine (1.7 mg/kg; Hospira, Inc.) was provided locally before midline incision. A craniotomy was made above the structure of interest, a glass micropipette containing retrobeads or AAV was lowered into the brain, and the injection was given at a rate of 2 nl/s (see below for experiment-specific details). OB injections were given at 1.5 mm anterior to the rhinal sinus, 1 mm lateral, and 1.5 mm ventral unless noted otherwise (Table 1). All OT injections were given at 1.5 mm anterior bregma, 1.2 mm lateral, and 4.8 mm ventral. Following injection, the micropipette was slowly withdrawn from the brain, the craniotomy sealed with wax, and the wound closed with Vetbond (3M Animal Care Products). The mice were returned to group housing immediately following surgery and were allowed to recover on a heating pad.

**Table 1.** Summary of all injections

Strain	*n*	Injected with	Injection site	Injection amount	Figure
C57BL/6J	4 (3 M, 1 F)	Retrobeads	OB (dispersed between 2400 and 800 μm ventral)	900 nl	2*A–C*, 3
C57BL/6J	2 M	Retrobeads	OB	200 nl	2*A–C*, 3
C57BL/6J	7 M	AAVretro-GFP	OB	200 nl	2*D*, 4*A–G*
C57BL/6J	2 M	AAVretro-GFP	OT	500 nl	4*H*
Ai9	6 M	AAVretro-Cre	OB	200 nl	2*E*, 5*A–G*
Ai9	1 M, 1 F	AAVretro-Cre	OT	500 nl	5*H*
Ai9	1 M, 2 sF	AAVretro-Cre	OB (1, 1.5, and 2 mm anterior the rhinal sinus, 1 mm lateral, 1.5 mm ventral)	200 nl × 3 sites = 600 nl total	6

Unless otherwise noted, OB injections were given at 1.5 mm anterior the rhinal sinus, 1 mm lateral, and 1.5 mm ventral, and OT injections were given at 1.5 mm anterior bregma, 1.2 mm lateral, and 4.8 mm ventral.

All experiments are summarized in Table 1. A total of 6 C57BL/6J mice (*n* = 5 male, *n* = 1 female) received unilateral injections of retrobeads in the OB. Each mouse received either 200 nl of retrobeads 1500 μm ventral to the surface (*n* = 2) or 900 nl evenly dispersed between 2400 and 800 μm ventral to the surface (*n* = 4). These differing strategies were employed to explore whether spatial targeting of the retrobeads in the OB impacted the outcome. We found that injecting 900 versus 200 nl resulted in a similar number of labeled cells in the AON (56.9 ± 12.8 vs 38.8 ± 6 cells; mean ± SEM) and PCX (39.9 ± 5.2 vs 34.7 ± 5.1 cells). A total of seven male C57BL/6J mice received 200-nl unilateral injections of AAVretro-GFP in the OB. An additional two male C57Bl/6J mice received a 500-nl injection of AAVretro-GFP in the OT. A total of six male Ai9 mice received 200-nl unilateral injections of AAVretro-Cre in the OB. Later, an additional three Ai9 mice (one male, two female) received three 200-nl OB injections each (at 1 mm, 1.5 mm, and 2 mm anterior to the rhinal sinus, 1 mm lateral, and 1.5 mm ventral), for a total of 600-nl AAVretro-Cre per mouse. An additional two Ai9 mice (one male, one female) received 500-nl unilateral injections of AAVretro-Cre in the OT.

### Perfusion and histology

All mice were overdosed with Fatal-plus (0.01 ml/g; Vortech Pharmaceutical, Ltd.) and perfused with 10 ml of cold saline followed by 15 ml of 10% PB formalin. Brains were stored in 10% formalin/30% sucrose (4°C) before sectioning.

All brains were frozen and alternate coronal sections were obtained with a sliding microtome at 40-μm thickness and stored floating in TBS with 0.03% sodium azide. Sections containing the OB, AON, anterior PCX (aPCX), and/or OT were rinsed in deionized water and mounted on slides using Fluoromount-G containing 4',6-diamidino-2-phenylindole (DAPI; Invitrogen). We selected 4–13 sections of each brain region for quantification, ensuring that sections spanned the anterior-posterior length of each region.

### Imaging

Brain areas of interest (OB, AON, aPCX, OT) were identified based on the atlas of Paxinos and Franklin (Paxinos and Franklin, 2000) and images acquired of the hemisphere ipsilateral to the OB injection. Imaging was performed with a Nikon Eclipse Ti2e fluorescent microscope at 20× magnification using a Nikon 16MP DS-Qi2 monochrome CMOS camera. Images of the OT following injections of AAV locally within the OT were acquired at 40× magnification. Images of the intact brain were taken using a 12 MP digital camera, and for fluorescence a Nikon AZ100 microscope at 1× magnification using a Photometrics CoolSNAP DYNO CCD camera. Image acquisition settings, including gain, exposure, and light intensity, were held constant across all images and samples within treatment conditions.

### Quantification and statistics

Successful injection was confirmed by observation of labeling in structures known to robustly innervate the OB (AON and aPCX; Price and Powell, 1970; Luskin and Price, 1983; Boyd et al., 2012; Markopoulos et al., 2012; Rothermel and Wachowiak, 2014; Padmanabhan et al., 2016). One out of seven AAVretro-GFP mice and two out of six AAVretro-Cre mice were excluded due to a lack of fluorescence anywhere in the brain, likely due to a mechanical failure of the injection.

**Figure 2.**
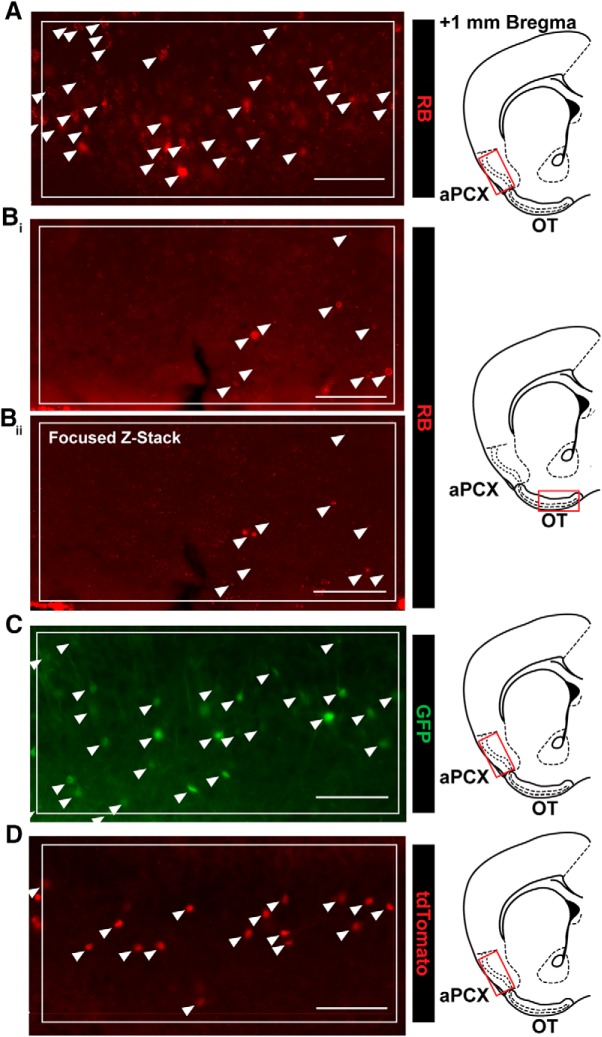
Representative results from semi-automated cell-counting procedures. ***A***, Representative results from semi-automated quantification of retrobead-labeled cells in aPCX. RB, retrobead. ***B_i_***, Representative results from semi-automated quantification of retrobead-labeled cells in the OT. ***B_ii_***, Same section as shown in ***B_i_***, as a focused z-stack, indicating that many cells counted were likely non-neuronal fluorescent puncta. Z-stack included six steps, with 4 μm between each step. ***C***, Representative results from semi-automated quantification of GFP-labeled cells in the aPCX following injection of AAVretro-GFP in the OB. ***D***, Representative results from semi-automated quantification of tdTomato-labeled cells in the aPCX following injection of AAVretro-Cre in the OB. Arrows indicate counted cells. Boxed region indicates ROI used for quantification. All scale bars = 100 μm.

An ROI bounding box (500 × 250 μm) was overlaid within each brain region of interest (aPCX, AON, OT), with effort made to hold the location of this bounding box constant across mice. Using semi-automated thresholding methods in NIS Elements (Nikon), we identified cells within these ROIs, allowing for an unbiased estimation of cell numbers. This first involved preprocessing of the image to decrease background fluorescence and thereby enhance contrast. Then cells were identified based on their fluorescence intensity (via threshold) and their size. Lastly, detected objects were post-processed based on their area of fluorescence, resulting in the elimination of objects too small to be cells (e.g., brightly labeled fibers in the Ai9 paradigm). Due to overt differences in cell-filling across our three tracing paradigms, these methods were optimized for each method individually, but an identical method was used across all images within a paradigm. Representative results from the semi-automated cell-counting procedures for each experimental paradigm are shown in Figure 2.

For each region analyzed, multiple sections were quantified for each mouse, with some variation in the number of sections quantified for each mouse (*n* = 16 mice, 6.75 ± 0.38 sections per brain region per mouse). This variation was due to occasional histologic imperfections (e.g., bubbles in the mounting medium or torn tissue) that precluded accurate quantification and resulted in the exclusion of some samples. To avoid overrepresenting mice for which more samples were analyzed, we calculated a mean for each region, and averaged these means across mice for each region. Any ROIs resulting in values exceeding two standard deviations outside the mean for that ROI were eliminated from all quantification and statistical analyses. This largely was applied toward retrobead-treated tissue wherein some sections had abundant fluorescence resulting from residual retrobeads collecting on the microtome blade and being deposited on latter sections.

## Results

### Retrobead labeling suggests lack of OT to OB innervation

**Figure 3.**
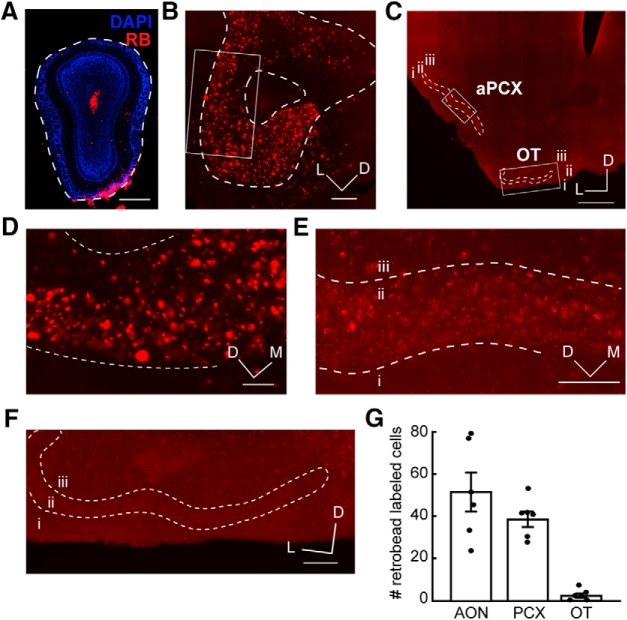
Retrobead injections to the OB indicate lack of OT to OB innervation. ***A***, OB injection site. Dotted line indicates the glomerular layer. Scale bar = 500 μm. ***B***, Retrobead labeling in the AON following OB injection. Box indicates region in ***D***. Scale bar = 200 μm. ***C***, aPCX and OT retrobead labeling. Boxes indicate regions in ***E***, ***F***. Scale bar = 500 μm. ***D***, Enhanced view of boxed AON region in ***B***. Scale bar = 100 μm. ***E***, Enhanced view of boxed aPCX region in ***C***. Scale bar = 100 μm. ***F***, Enhanced view of boxed OT region in C. Scale bar = 100 μm. ***G***, Estimated number of retrobead-labeled cells across all three brain regions. Each point represents one animal’s mean; *n* = 6 mice, four to six sections (4.78 ± 0.19; mean ± SEM) each. i.–iii., layers 1–3; D, dorsal; M, medial; L, lateral.

In our first experimental paradigm (Fig. 1*A*), we injected retrobeads unilaterally in the OB of C57BL/6J mice (Fig. 3*A*). At the site of injection, retrobeads are endocytosed and retrogradely transported to the soma, resulting in an accumulation of retrobeads and fluorescently labeled soma. As expected, this resulted in fluorescent soma in the AON and aPCX, two structures known to innervate the OB (Price and Powell, 1970; Luskin and Price, 1983; Boyd et al., 2012; Markopoulos et al., 2012; Rothermel and Wachowiak, 2014; Padmanabhan et al., 2016; Fig. 3*B–E*). Unexpectedly, our automated cell counting returned only a few positive OT values, from just a subset of tissue sections (Fig. 3*F*,*G*). On visual inspection of the counted “cells” in the OT, and inspection of z-stack images, we determined that these were non-neuronal fluorescent puncta (Fig. 2*B*), likely residual retrobeads deposited on the tissue during sectioning.

### Viral tracing further supports lack of centrifugal OT to OB input

**Figure 4.**
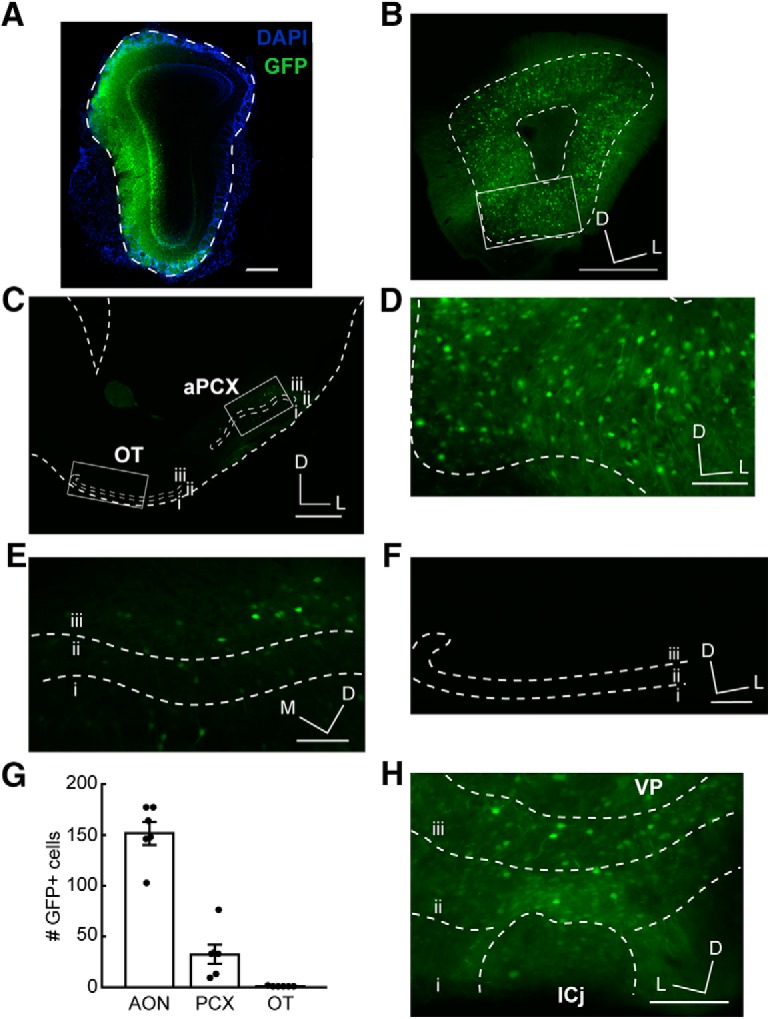
AAVretro-GFP tracing further supports lack of OT to OB innervation. ***A***, OB injection site. Dotted line indicates the glomerular layer. Scale bar = 500 μm. ***B***, AON GFP labeling following injection of AAVretro-GFP in the OB. Box indicates region in Figure 3*D*. Scale bar = 500 μm. ***C***, aPCX and OT GFP labeling following injection of AAVretro-GFP in the OB. Boxes indicate regions in Figure 3*E*,*F*. Scale bar = 500 μm. ***D***, Enhanced view of boxed AON region in B. Scale bar = 100 μm. ***E***, Enhanced view of boxed aPCX region in ***C***. Scale bar = 100 μm. ***F***, Enhanced view of boxed OT region in ***C***. Scale bar = 100 μm. ***G***, Quantification of GFP-labeled cells across all three brain regions. Each point represents one animal’s mean; *n* = 6 mice, 4–12 sections (6.89 ± 0.54; mean ± SEM) each. ***H***, GFP labeling in the OT following injection of AAVretro-GFP in the OT; *n* = 2 mice. VP, ventral pallidum; ICj, islands of Calleja; ICj, borders were approximated based on DAPI staining. Scale bar = 100 μm. i.–iii., layers 1–3; D, dorsal; M, medial; L, lateral.

Given the surprising lack of OT fluorescence observed in the retrobead experiment, we sought to confirm our findings by using a viral labeling technique (Fig. 1*B*) to rule out the possibility of a false negative result. We unilaterally injected C57BL/6J mice in the OB with AAVretro-GFP to label neurons projecting to the OB (Fig. 4*A*). This virus drives GFP expression under control of the human synapsin promoter, and is thus capable of labeling any neuronal cell type (Kügler et al., 2003), including medium spiny neurons of the ventral striatum (McLean et al., 2014) of which the OT is largely comprised. With this independent paradigm, we observed many GFP-labeled neurons in the AON and aPCX (Fig. 4*B–E*), indicating that we were successful in labeling neurons that centrifugally innervate the OB. In contrast, we again observed a lack of fluorescent neurons in the OT (Fig. 4*B–G*).

To ensure that the virus we used is capable of infecting and driving GFP expression in OT neurons, we unilaterally injected the OT directly with AAVretro-GFP in a separate cohort of C57BL/6J mice. We observed GFP expression in OT neurons across all cell layers of the OT (Fig. 4*H*), including in the islands of Calleja, suggesting that the lack of GFP expression in the OT following AAVretro-GFP injection into the OB is not due to an issue of AAV tropism for OT neurons.

### Cre-dependent labeling strategy confirms lack of OT to OB pathway

**Figure 5.**
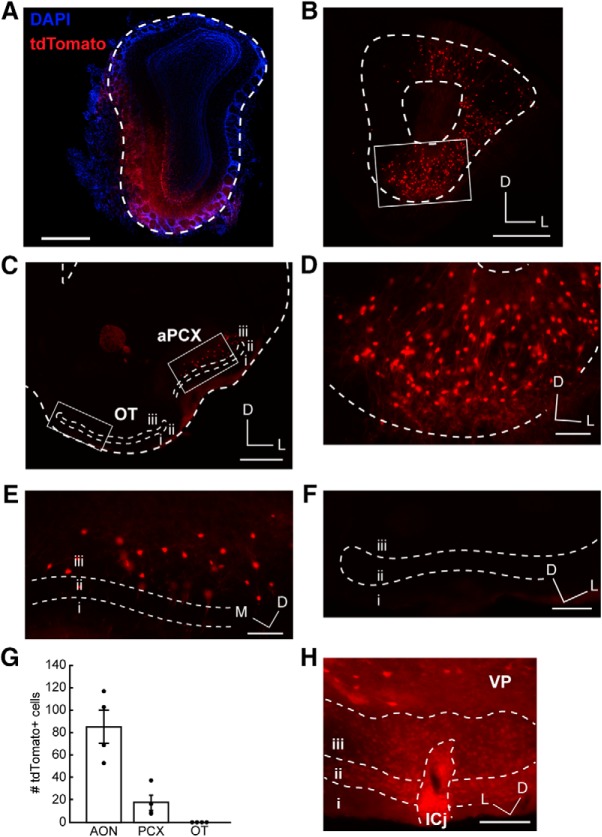
AAVretro-Cre tracing in Ai9 reporter mouse verifies lack of OT to OB innervation. ***A***, OB injection site. Dotted line indicates the glomerular layer. Scale bar = 500 μm. ***B***, AON tdTomato labeling following injection of AAVretro-Cre in the OB of Ai9 mouse. Box indicates region in Figure 4*D*. Scale bar = 500 μm. ***C***, aPCX and OT tdTomato labeling following injection of AAVretro-Cre in the OB of Ai9 mouse. Boxes indicate regions in Figure 4*E*,*F*. Scale bar = 500 μm. ***D***, Enhanced view of boxed AON region in ***B***. Scale bar = 100 μm. ***E***, Enhanced view of boxed aPCX region in ***B***. Scale bar = 100 μm. ***F***, Enhanced view of boxed OT region in ***C***. Scale bar = 100 μm. ***G***, Quantification of tdTomato-labeled cells across all the three brain regions. Each point represents one animal’s mean; *n* = 4 mice, 5–13 sections/mouse (9.5 ± 0.68; mean ± SEM). ***H***, OT neurons labeled following injection of AAVretro-Cre in the OT of Ai9 mouse; *n* = 2 mice. VP, ventral pallidum; ICj, islands of Calleja. ICj borders were approximated based on DAPI staining. Scale bar = 100 μm. i.–iii., layers 1–3; D, dorsal; M, medial; L, lateral.

Finally, we employed a third strategy in an attempt to label this pathway. We used Ai9 reporter mice, which robustly express tdTomato in a Cre-dependent manner (Madisen et al., 2010). We unilaterally injected AAVretro-Cre (which drives Cre expression under control of the Ef1α promoter and is thus capable of driving Cre expression in any mammalian cell) into the OB (Figs. 1*C*, 5*A*). This strategy resulted in robust tdTomato expression in structures known to innervate the OB, including the aPCX and AON (Fig. 5*B–E*). We observed a complete and striking lack of cells in the OT (Fig. 5*F*), which was in stark contrast to the plentiful cells observed in the AON and the aPCX (Fig. 5*G*).

Once again, to ensure that the virus we used is capable of infecting OT neurons, we injected AAVretro-Cre directly into the OT of a separate cohort of Ai9 mice. As for AAVretro-GFP (Fig. 4*H*), this approach yielded fluorophore-expressing cells throughout the OT (Fig. 5*H*).

**Figure 6.**
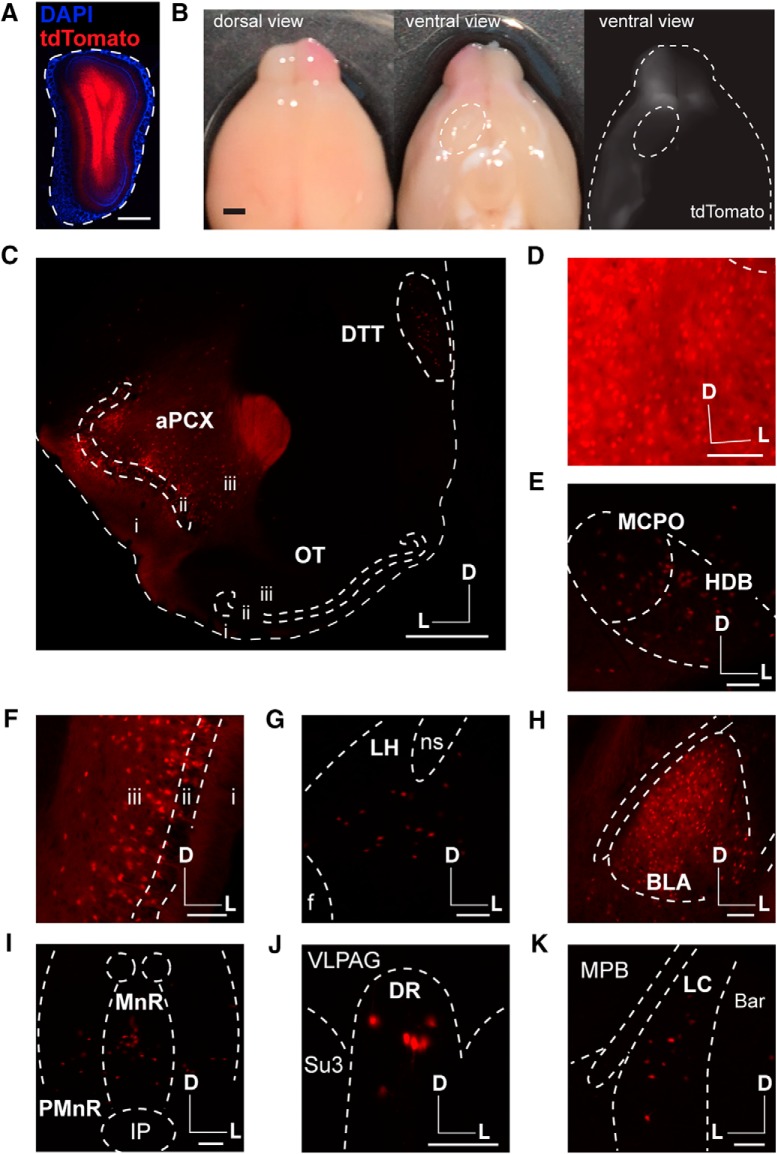
Multiple injections of AAVretro-Cre in the OB of Ai9 reporter mouse reveals labeling in numerous OB-projecting structures, but not the OT. ***A***, OB injection site. Dotted line indicates the glomerular layer. Scale bar = 500 μm. ***B***, Intact brain following multiple injections of AAVretro-Cre in the OB of Ai9 reporter mouse shows strong tdTomato labeling in one OB and the PCX, but not the OT. Dotted line indicates the OT. Scale bar = 1 mm. ***C***, aPCX and OT tdTomato labeling following injection of AAVretro-Cre in the OB of Ai9 mouse. Scale bar = 500 μm. ***D*–*K***, tdTomato labeling in many regions following injection of AAVretro-Cre in the OB of Ai9 mouse. All scale bars = 100 μm. ***D***, AON. ***E***, Horizontal diagonal band of Broca (HDB) and magnocellular preoptic nucleus (MCPO). ***F***, Posterior PCX (pPCX). ***G***, Lateral hypothalamus (LH). ***H***, Basolateral amygdala (BLA). ***I***, Median raphe nucleus (MnR) and paramedian raphe nucleus (PMnR). ***J***, Dorsal raphe nucleus (DR). ***K***, Locus coeruleus (LC). f, fornix; ns, nigrostriatal bundle; IP, interpeduncular nucleus; VLPAG, ventrolateral periaqueductal gray; Su3, supraoculomotor cap; MPB, medial parabrachial nucleus; Bar, Barrington’s nucleus. i.–iii., layers 1–3; D, dorsal; M, medial; L, lateral; *n* = 3 mice.

**Figure 7.**
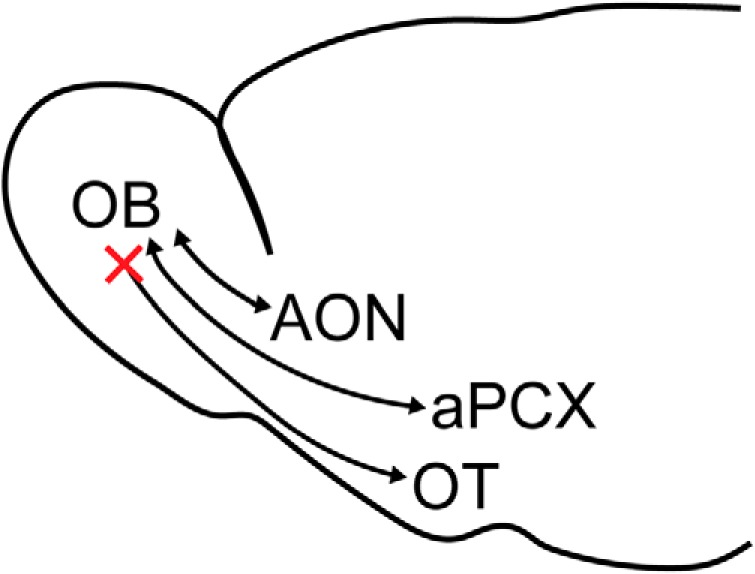
Revised model for centrifugal inputs to the OB. The AON, aPCX, and OT receive input from the OB. The AON and aPCX send centrifugal input back to the OB, but based on our data, the OT does not.

Finally, we wanted to ensure that we were not inadvertently missing any regions of the OB that may be the target of OT centrifugal input. Indeed, it is possible OT projections to the OB may innervate notably small portions of the OB, which might be missed with the single bolus injections largely employed in the previous approaches (although those injections often did span OB cell layers (Figs. 4*A*, 5*A*). We also sought to ensure our retrograde OB injection approaches were capable of labeling neurons in structures other than just the aPCX and AON. To accomplish these goals, we unilaterally injected an additional cohort of Ai9 mice with AAVretro-Cre at three sites along the anterior-posterior axis in the same OB and collected tissue sections throughout the entire brain (in contrast to the previous paradigms wherein we only collected forebrain tissue and analyzed the aPCX, AON, and OT). This strategy resulted in robust tdTomato expression throughout the entire OB (Fig. 6*A*), which was evident even on looking at the intact brain (Fig. 6*B*). tdTomato expression appeared absent within the OT when viewing the ventral side of the intact brain with the naked eye and under epifluorescence (Fig. 6*B*). After sectioning, we again did not observe fluorescent neurons in the OT (Fig. 6*C*), in contrast to significant labeling in many other structures known to innervate the OB, including the aPCX (Fig. 6*C*), dorsal tenia tecta (Shipley and Adamek, 1984; Fig. 6*C*), AON (Fig. 6*D*), horizontal diagonal band of Broca (Ichikawa and Hirata, 1986; Devore and Linster, 2012; Rothermel et al., 2014; Fig. 6*E*), magnocellular preoptic area (Carson, 1984; Fig. 6*E*), posterior PCX (Fig. 6*F*), lateral hypothalamus (Shipley and Adamek, 1984; Fig. 6*G*), basolateral amygdala (Fig. 6*H*), median and dorsal raphe nuclei (Steinfeld et al., 2015; Brunert et al., 2016; Fig. 6*I*,*J*), and locus coeruleus (Shipley et al., 1985; McLean et al., 1989; Fig. 6*K*). Because this approach resulted in very thorough labeling of the OB, as well as labeling in many areas known to innervate the OB, these results provide strong evidence that the OT does not send centrifugal projections to the OB (Fig. 7). Together with our results from 16 mice across three different quantitative experimental approaches (six retrobead, six AAVretro-GFP, and four AAVretro-Cre; Figs. 3–6), we observed no OB-projecting OT neurons, providing strong support for our conclusion that the OT does not project to the OB.

## Discussion

Here, we demonstrate a lack of input from the OT to the OB. While we initiated this study with the goal of investigating the anatomic organization and physiologic role of this reported pathway (Shafa and Meisami, 1977), our results ultimately lead us to the conclusion that it does not exist. To minimize the chance of a false negative result, we used three independent, widely-used tracing methods, each of which yielded congruent results. Our use of a retrograde labeling strategy eliminated the possibility that we may have introduced non-specific labeling due to off-target effects that can accompany anterograde tracing methods when the structure of interest is difficult to target due to small size or irregular shape, like the OT. The regions of interest were chosen in the same portion of the structures across all animals within each experiment to ensure fair sampling. Further, we set rigorous standards for sample exclusion, excluding only samples that showed no labeling in regions with very well-characterized, dense projections to the OB.

In only one of our experimental paradigms, the retrobead paradigm, did our semi-automated cell counting return positive cell counts in the OT. For some of these instances, we performed z-stack imaging which indicated that the cells counted were actually non-neuronal fluorescent puncta. It seems those artifacts were residual retrobeads that were deposited by the microtome blade onto subsequent sections (an issue we observed and noted during the tissue sectioning). This issue may also contribute to the fact that we counted slightly more cells in the aPCX in the retrobead experiments compared to the AAV experiments. In support of OT cells not innervating the OB, the results we obtained using the two viral tracing techniques indicated a complete absence of cells in the OT.

We did observe some slight differences in the numbers of cells labeled by each tracing method. For example, we observed slightly more retrobead-labeled neurons in the aPCX compared to either viral tracing method, which were likely due to differences in the efficiency of endocytosis and transport of the retrobeads compared to virally-mediated GFP or Cre expression. Importantly, each method indicated the same pattern of labeling, with the densest labeling in the AON, followed by the aPCX, and no labeling in the OT. Overall, the slight differences we observed with each technique highlight the importance of using multiple strategies in our attempt to identify OB-projecting OT neurons.

The failure of all three of our primary strategies to label OT neurons projecting to the OB provides strong support for our conclusion that this population does not exist. This is further strengthened by the outcomes of our follow-up experiments, wherein we injected multiple boluses of AAVretro-Cre widely throughout the OB of Ai9 mice. This revealed, as expected, tdTomato expression in nearly one dozen brain structures with known innervation of the OB, but not the OT (Fig. 6).

Our results are surprising, given the previous reports that suggested the existence of this pathway. In one study (Shafa and Meisami, 1977), the authors injected HRP into the rat OB. The authors concluded that there was some “light” labeling in the OT but did not provide quantitative analysis, and only presented minimal primary data. Two studies (Heimer, 1968; Gervais, 1979) revealed degeneration in the OB following lesions to the OT, suggesting that the OT sends feedback to the OB. The Heimer study (Heimer, 1968) mentions the difficulty of lesioning the OT without damaging neighboring structures, suggesting off-target effects (namely, damage beyond the OT) may contribute to their result. The lesioning strategy used in the Gervais paper (Gervais, 1979) is subject to the same caveat. Indeed, some of their OT lesions resulted in damage to part of the lateral olfactory tract (which provides input to all olfactory cortices), thus introducing the possibility of highly non-specific effects. In both cases, even if lesions were perfectly confined to the OT, OB degeneration could result from the loss of an indirect connection to the OB via a third structure, rather than the loss of a monosynaptic connection.

A more recent study (Zhang et al., 2017b) used viral anterograde and retrograde tracing techniques to study the inputs and outputs of the OT. With an anterograde AAV injection into the OT, the authors reported labeling throughout the OB, with the majority of fluorescence localized within the glomerular layer of the OB. As with the lesioning experiments discussed above, it can be quite difficult to exclusively target an injection to the mouse OT without any leak into surrounding areas (like the aPCX which does project to the OB). Here, we avoid this caveat by using three independent retrograde labeling strategies, each of which indicates a lack of projection from the OT to the OB.

It is well established, and further supported by our data, that the AON and PCX provide centrifugal innervation to the OB (Price and Powell, 1970; Luskin and Price, 1983; Boyd et al., 2012; Markopoulos et al., 2012; Rothermel and Wachowiak, 2014; Padmanabhan et al., 2016). However, our results indicate a lack of direct input from the OT to the OB. What might this indicate about the role of the OT within the olfactory system? OT neurons receive input from the OB and encode odors in a cortical-like manner, suggesting a role in odor processing (Wesson and Wilson, 2010). Further, these responses are modulated by attention (Zelano et al., 2005; Carlson et al., 2018), and it has previously been speculated that the OT may play a role in top-down, state-dependent modulation of OB activity (Gervais, 1979; Wesson and Wilson, 2011). Ultimately, our data lead us to conclude that it is very unlikely the OT directly modulates OB activity, suggesting that state-dependent modulation of OB activity must result from other sources of input (e.g., AON, aPCX), or indirect input from the OT by means of other structures. Of course, our data do not rule out the highly likely possibility that the OT may indirectly, via di- or even tri-synaptic connections, influence OB activity through its projections to regions that do innervate the OB, like the aPCX or even AON. Future work could attempt to address this possibility using optogenetic and electrophysiological methods.

## Synthesis

Reviewing Editor: Cheryl Sisk, Michigan State University Decisions are customarily a result of the Reviewing Editor and the peer reviewers coming together and discussing their recommendations until a consensus is reached. When revisions are invited, a fact-based synthesis statement explaining their decision and outlining what is needed to prepare a revision will be listed below. The following reviewer(s) agreed to reveal their identity: Claire Cheetham, James Cherry.

The authors were receptive to the critiques of the original manuscript, and have done several additional groups of animals to address these critiques. There are a few additional comments on the figures and the statistics that should be addressed:

Fig 2: could use an illustration of where the images were taken from.

Fig 3A and 4A and 6A: the outline of the OB should be shown (especially for fig 3A) as it is difficult to see. Also the DAPI-labeling in these figures is especially difficult to see-can these by lightened at all?

Fig 3: the caption appears to refer to the wrong images for some of the boxes.

Fig 4C: some additional tracing of the outline of the brain might be useful as the image is extremely dark.

lines 247-249. If the observation is that the OT does not send input to the OB, then the use of statistics here is misleading. To say that there were significantly more cells labeled in the AON and PCX than in the OT suggests that there were some cells labeled in the OT, just not as many. This is very different from what the authors are concluding, which is that there is no centrifugal projection from the OT to the OB. I understand the desire to put a statistical stamp on the result, but it should be emphasized that there were no labeled cells in the OT. Notably, the authors do address this issue in lines 234-236 for the retrobeads results, where it was stated that the few counted cells in the OT were deemed to be artifactual.

Related to this issue, the new statistical analysis is flawed; although the non-parametric tests are a better choice, the authors went from using a paired t-test (dependent samples) to a Wilcoxon M-W test, which is for independent samples. They should have used the Wilcoxon signed-ranks test. With n=4 subjects (which was the sample size for the AAV-Cre study shown in Fig 5G) it is not possible to get a significant p-value with the Wilcoxon s-r test, no matter what the numbers are. However, comparing whether brain areas differ in the number of labeled cells is not really the point of the study, so the statistical significance (or lack thereof) using such tests is not particularly informative. The AAV-Cre results do confirm the findings of the first two methods, even if on their own they may not be amenable to statistical treatment. Ultimately it might just be better to take the results of all 3 approaches together (with 6, 6, and 4 subjects total), and state that out of 16 subjects there were no labeled cells in the OT.

## Author Response

Synthesis Statement for Author (Required)

Reviewers agreed that the question of whether there are reciprocal connections between the MOB and the OT is of interest, and that confirmation of the lack of an OT-MOB projection would be an important finding. However, both reviewers raise significant concerns about methodology and analyses that must be addressed for the results to make a convincing case for the absence of an OT-MOB projection. In particular, it is critical to show that the retroAAVs used in the study are able to infect OT neurons before concluding that the AVV studies provide evidence of no OT-MOB projection. In addition, please provide a stronger rationale for the approach of a single injection at a single MOB site in the majority of mice (i.e., is this approach sufficient to capture centrifugal terminals from the OT, especially if they are sparse?). Finally, more information on the statistical analyses and methods for detecting and counting labeled OT cells is needed for better evaluation of the results and interpretation.

We thank the reviewers for their time in providing thoughtful comments and suggestions to improve this manuscript. In order to address the concerns raised, we have completed additional experiments. First, we demonstrated that each of the viruses we used are capable of infecting OT neurons by injecting them directly into the OT (see point 1 below). In order to address the concern that single injections into the OB might not be sufficient to capture sparse inputs to the OB, we injected AAVretro-Cre at three different anterior-posterior sites of the OB in a new cohort of Ai9 mice (see points 2 and 3 below). The results of those experiments allowed us to uphold our conclusions. We have also included more information about our cell counting procedure, and have incorporated a new figure showing examples of counted cells using our methods, including examples of false positives counted in the retrobead experiments (see points 5 and 6 below). Due to the addition of new experiments, we also included a new table which summarizes each injection completed and includes information about the injection location, amount of virus, number of mice, Figure #, etc. Please note that all line numbers referenced below refer to the “article file” (which has insertions indicated in red).

Major comments

1. While the absence of labeled cells in the OT in the retro-AAV experiments is convincing, this could be because the retro-AAVs used are not able to infect OT neurons, whereas they do infect AON and PCX neurons. Different AAV serotypes have tropism for different subsets of neurons; the retro-AAV used here is relatively new and, to my knowledge, has not previously been employed to label OT neurons. The authors should perform control experiments with both retroAAVs, to demonstrate that direct injection into OT does label neurons there. In general, the authors should provide more explanation of the rationale for choosing the 3 retrograde labeling methods, particularly for the mice receiving AAVs. It should be mentioned which cells were expected to be labeled and why. In particular for the Ai9 mice, in what cells is the reporter capable of being expressed (i.e., when cre recombinase is present)? Similarly, do hSyn and Ef1α drive expression of cre recombinase in every infected cell?

To address the issue of viral tropism, we delivered injections of AAVretro-GFP or AAVretro-Cre directly to the OT of C57BL/6J or Ai9 mice, respectively, and observed that these viruses are both capable of infecting OT neurons. This data is now shown in Fig. 4H and Fig. 5H and we have included the following text in the Results section:

Lines 246-251 “To ensure that the virus we used is capable of infecting and driving GFP expression in OT neurons, we unilaterally injected the OT directly with AAVretro-GFP in a separate cohort of C57BL/6J mice. We observed GFP expression in OT neurons across all cell layers of the OT (Fig. 4H), including in the islands of Calleja, suggesting that the lack of GFP expression in the OT following AAVretro-GFP injection into the OB is not due to an issue of AAV tropism for OT neurons.”

Lines 262-265 “Once again, to ensure that the virus we used is capable of infecting OT neurons, we injected AAVretro-Cre directly into the OT of a separate cohort of Ai9 mice. As for AAVretro-GFP (Fig 4H), this approach yielded fluorophore-expressing cells throughout the OT (Fig. 5H).”

We have also included some text in the Results section about the cell types that should be labeled by each viral strategy we used:

Lines 238-241 “This virus drives GFP expression under control of the human synapsin promoter, and is thus capable of labeling any neuronal cell type (Kügler et al., 2003), including medium spiny neurons of the ventral striatum (McLean et al., 2014) of which the OT is largely comprised.”

Lines 256-258 “We unilaterally injected AAVretro-Cre (which drives Cre expression under control of the Ef1α promoter and is thus capable of driving Cre expression in any mammalian cell) into the OB (Fig. 1C, Fig. 5A).”

2. To provide a more convincing demonstration that the OT is devoid of cells that project to the OB, more brain regions with known centrifugal input to the MOB should be examined. In addition to the AON and PC, what about the cholinergic inputs from the HDB, serotonergic innervation from both the median and dorsal raphe, noradrenergic inputs from the LC, etc? If all of these regions except for the OT are shown to be labeled by the methods the authors have used, then the case is strengthened. As it stands, the primary finding of the study (or lack thereof) depends on the retrobeads, and the emphasis on the AAV results needs to be toned down unless it is shown that the viruses label more sites known to project to the MOB than just the AOB and PC. Analysis of sections containing these additional brain regions from these experiments would address this concern.

For our original submission, we regrettably did not collect sections containing the LC, raphe, or others. To address this we have performed injections of AAVretro-Cre into the OB of additional Ai9 mice. This experiment is described in the Methods section:

Lines 149-153: “Later, an additional 3 Ai9 mice (1 male, 2 female) received 3 200nL OB injections each (at 1mm, 1.5mm, and 2mm anterior to the rhinal sinus, 1mm lateral, and 1.5mm ventral), for a total of 600nL AAVretro-Cre per mouse.”

The results of this experiment are included in a new figure (Fig. 6) and described in the Results section:

Lines 266-291: “Finally, we wanted to ensure that we weren't inadvertently missing any regions of the OB that may be the target of OT centrifugal input. Indeed, it is possible OT projections to the OB may innervate notably small portions of the OB, which might be missed with the single bolus injections largely employed in the previous approaches (although those injections often did span OB cell layers (Figs 4A & 5A). We also sought to ensure our retrograde OB injection approaches were cable of labeling neurons in structures other than just the aPCX and AON. To accomplish these goals, we unilaterally injected an additional cohort of Ai9 mice with AAVretro-Cre at three sites along the anterior-posterior axis in the same OB and collected tissue sections throughout the entire brain (in contrast to the previous paradigms wherein we only collected forebrain tissue and analyzed the aPCX, AON, and OT). This strategy resulted in robust tdTomato expression throughout the entire OB (Fig. 6A), which was evident even upon looking at the intact brain (Fig. 6B). tdTomato expression appeared absent within the OT when viewing the ventral side of the intact brain with the naked eye and under epifluorescence (Fig. 6B). After sectioning, we again did not observe fluorescent neurons in the OT (Fig. 6C), in contrast to significant labeling in many other structures known to innervate the OB, including the aPCX (Fig. 6C), dorsal tenia tecta (Shipley and Adamek, 1984) (Fig. 6C), AON (Fig. 6D), horizontal diagonal band of Broca (Devore and Linster, 2012; Ichikawa and Hirata, 1986; Rothermel et al., 2014) (Fig. 6E), magnocellular preoptic area (Carson, 1984) (Fig. 6E), posterior PCX (Fig. 6F), lateral hypothalamus (Shipley and Adamek, 1984) (Fig. 6G), basolateral amygdala (Fig. 6H), median and dorsal raphe nuclei (Brunert et al., 2016; Steinfeld et al., 2015) (Fig. 6I-J), and locus coeruleus (McLean et al., 1989; Shipley et al., 1985) (Fig. 6K). Because this approach resulted in very thorough labeling of the OB, as well as labeling in many areas known to innervate the OB, these results provide strong evidence to support our conclusion that the OT does not send centrifugal projections to the OB (Fig. 7).”

3. A major concern is that in 2 of the groups of mice apparently only one injection was made at one site in the MOB (1500 um ventral to the surface- why was this coordinate chosen?). While multiple injections were given using the retrobeads in the 3rd group of mice, it still can't be assumed that all centrifugal terminals had access to the tracer. On this point, the figures should include photos of the MOB injection sites. Although complete labeling of all terminals is unlikely, the description in the manuscript provided no indication of the expected extent to which centrifugal inputs might be labeled or why it was thought that one or even several injections at one A-P site was sufficient. In the group with multiple injections there was no mention of how many animals received this treatment. While the authors acknowledge that spatial targeting was a possibility, they addressed this only in the retrobeads group and it is not even clear how many of the animals in this group received the multiple injections.

We have now included representative OB images from each experiment showing the injection site (Fig. 3A, Fig. 4A, Fig. 5A, Fig. 6A). We have also clarified that 4 out of 6 retrobead injected mice received multiple injections, and compared the quantified cells between these two groups. This is now mentioned in the Methods section:

Lines 139-146: “A total of 6 C57BL/6J mice (n=5 male, n=1 female) received unilateral injections of retrobeads in the OB. Each mouse received either 200nL of retrobeads 1500μm ventral to the surface (n=2) or 900nL evenly dispersed between 2400μm and 800μm ventral to the surface (n=4). These differing strategies were employed to explore whether spatial targeting of the retrobeads in the OB impacted the outcome. We found that injecting 900 nL vs. 200 nL resulted in a similar number of labeled cells in the AON (56.9{plus minus}12.8 vs. 38.8{plus minus}6 cells, mean {plus minus} SEM) and PCX (39.9{plus minus}5.2 vs. 34.7{plus minus}5.1 cells, mean {plus minus} SEM).”

Based on this, we believed that by using a single (relatively large) injection, we would capture a representative sample of all centrifugal input to the OB, but we understand the concern about this approach. We have now included new experiments wherein we injected AAVretro-Cre at three A-P sites in the OB of Ai9 mice (see point 2 above). This resulted in very thorough labeling throughout the entire OB (Fig. 6A-B), yet still no labeling in the OT (Fig. 6C).

4. The authors note that their data are normally distributed, but do not state how they tested this. The authors do not state that they tested for equal variance, the second assumption that must be met in order to use parametric statistical tests. From their graphs, it is unlikely that all data sets have equal variance, especially given the small numbers in their OT data sets. Normality and equal variance testing should be performed and if data fail one or more of these tests, non-parametric tests should instead be used.

We have now corrected this issue, and the statistical table is updated with the appropriate tests. This is explained in the Methods section:

Lines 211-214 “Each dataset was tested for normality using the Shapiro-Wilk test. For each comparison, we tested for equal variances using the two-sample F-test. For all comparisons made, the variances were not equal, and significance was determined using the Wilcoxon-Mann-Whitney test with a significance level (p) set at 0.05 (Table 2).”

5. The authors state that variable numbers of sections (4-6 for Fig.2, 4-12 for Fig.3, 5-13 for Fig.4) were used for quantification of the number of labeled cells, from which “mean cell count per animal” was determined. It is unclear what this number means: is it the mean of the number of cells within the region of interest per section? Furthermore, why was the number of sections quantified so variable between animals? I am concerned that the use of this metric could bias the results. I would suggest that reporting cell density per animal, encompassing data from a consistent number of sections per animal, would provide a more accurate metric of retrograde labeling.

We have clarified our quantification methods, including the reason for differences in the number of sections quantified for each animal, in the Methods section:

Lines 200-206 “For each region analyzed, multiple sections were quantified for each mouse, with some variation in the number of sections quantified for each mouse (n=16 mice, 6.75 {plus minus} 0.38 sections per brain region per mouse, mean {plus minus} SEM). This variation was due to occasional histological imperfections (e.g. bubbles in the mounting medium or torn tissue) that precluded accurate quantification and resulted in the exclusion of some samples. In order to avoid over-representing mice for which more samples were analyzed, we calculated a mean for each region, and averaged these means across mice for each region.”

Our quantification of means of cells / region of interest within animals allows for us to know the metric does NOT “bias” the results depending upon section numbers as raised by this reviewer, as for instance would be an issue if we were to sum the number of cells and use a 'total number of cells' metric. We have also clarified this in the figure legends, by replacing “mean cell count per animal” with “each point represents one animal's mean”.

6. Cell counting methodology: the authors state that semi-automatic thresholding and size were used to detect cells, and that these parameters were optimized for each labeling method. I understand that different thresholding may be required for each labeling method. However, I would like to see additional information on the thresholding method(s) used, and more importantly, examples of what was detected as a cell in the images. Without this, I cannot determine whether the cell detection method appears accurate.

We have incorporated a new figure (Fig. 2) showing representative results of our automated cell-counting method for all three approaches. This figure is referenced in the Methods section, where we have also included more information about the semi-automated quantification process:

Lines 191-199 “This first involved preprocessing of the image to decrease background fluorescence and thereby enhance contrast. Then cells were identified based upon their fluorescence intensity (via threshold) and their size. Lastly, detected objects were post-processed based on their area of fluorescence, resulting in the elimination of objects too small to be cells (e.g., brightly labeled fibers in the Ai9 paradigm). Due to overt differences in cell-filling across our three tracing paradigms, these methods were optimized for each method individually, but an identical method was used across all images within a paradigm. Representative results from the semi-automated cell-counting procedures for each experimental paradigm are shown in Figure 2.”

7. It is notable that 2/6 mice in one group and 1/7 mice in another were excluded because there was no labeling in the PC or AON. Unless this was due to a mechanical dysfunction (e.g., clogged needle), it suggests that there was at least some variability in the methods such that labeling doesn't occur even in the areas with the heaviest centrifugal input. In subjects with weaker labeling of the AON and PC, one could imagine that structures with modest or weak MOB input (the OT?) fail to label at all. It would be very helpful for the authors to explain why they think the injections failed to result in any labelling to better understand this issue.

Indeed, these mice were excluded due to some sort of mechanical failure. This is now clarified in the Methods section.

Lines 184-186 “1 out of 7 AAVretro-GFP mice and 2 out of 6 AAVretro-Cre mice were excluded due to a lack of fluorescence anywhere in the brain, likely due to a mechanical failure of the injection.”

Additionally, in order to address the concern that modest/weak inputs to the OB might not be labeled with our initial method, we performed additional injections at three anterior-posterior sites (Fig. 6, also see response to point 3).

8. Results line 200, Discussion lines 241-244: retrobead-labeling in OT. Because the reason for seeing some labeling in OT by retrobeads is not explained until the Discussion, the reader may miss the reason for this. I would also like to see an example of a z-stack image showing that only isolated retrobeads, rather than clearly retrobead-labeled cells were found here; this would greatly increase confidence in this result. Please include such an image in Fig.2 and an explanation of this in the relevant Results section.

We have now included a mention of these false positive results in the Results section.

Lines 230-232 “Upon visual inspection of the counted ”cells“ in the OT, we determined that these were non-neuronal fluorescent puncta (Fig. 2B) - likely residual retrobeads deposited on the tissue during sectioning.”

Additionally, we have included a figure panel (Fig. 2B-C), including a focused z-stack image, showing a representative fluorescent puncta/artifacts which were counted as “cells” in our analysis. This kind suggestion will help the readers understand these are truly false-positives from this one method.

Minor comments

9. There is no discussion of why much lower numbers of PCX cells were labeled by both of the retro-AAV strategies compared to the retrobead strategy. Please include some discussion; the issue of retro-AAV tropism may also be relevant here.

We have now included in the Discussion section some discussion of this issue:

Lines 317-324 “We did observe some slight differences in the numbers of cells labeled by each tracing method. For example, we observed slightly more retrobead-labeled neurons in the aPCX compared to either viral tracing method, which were likely due to differences in the efficiency of endocytosis and transport of the retrobeads compared to virally-mediated GFP or Cre expression. Importantly, each method indicated the same pattern of labeling, with the densest labeling in the AON, followed by the aPCX, and no labeling in the OT. Overall, the slight differences we observed with each technique highlight the importance of utilizing multiple strategies in our attempt to identify OB-projecting OT neurons.”

10. Lines 130-131: there are words missing- the pipette wasn't lowered at 2nL/s

Thank you for catching this - it is corrected.

11. Line 151: please provide the orientation of the sections

The sections were coronal; this is now mentioned in the methods section.

12. Line 264: the term 'disynaptic', rather than 'bisynaptic' is typically used

Thank you for catching this - it is corrected.

13. Line 271: Fibers of passage would need to be going somewhere; it is not clear why fibers would enter the olfactory bulb only to pass through, or where they would be going, given its anatomical location.

This statement has now been omitted.
